# The development of early ascites is associated with shorter overall survival in patients with hepatocellular carcinoma treated with drug-eluting embolic chemoembolization

**DOI:** 10.1186/s12876-020-01307-x

**Published:** 2020-06-01

**Authors:** María Pipa-Muñiz, Susana Sanmartino, Alicia Mesa, Carmen Álvarez-Navascués, Maria-Luisa González-Diéguez, Valle Cadahía, José-Eduardo Rodríguez, Florentino Vega, Manuel Rodríguez, Serafin-Marcos Costilla-García, María Varela

**Affiliations:** 1 Division of Gastroenterology and Hepatology, Hospital de Cabueñes, Gijón, Spain; 2 Radiology Department, Hospital Universitario Central de Asturias, Oviedo, Spain; 3 Liver Unit, Hospital Universitario Central de Asturias, 33011 Oviedo, Spain; 4 Universidad de Oviedo, Oviedo, Spain

**Keywords:** Hepatocellular carcinoma, DEB-TACE, Ascites, Response, Survival

## Abstract

**Background:**

A single-centre cohort study was performed to identify the independent factors associated with the overall survival (OS) of hepatocellular carcinoma (HCC) patients treated with transarterial chemoembolization with drug-eluting beads (DEB-TACE).

**Methods:**

A total of 216 HCC patients who underwent DEB-TACE from October 2008 to October 2015 at a tertiary hospital were consecutively recruited. The analysis of prognostic factors associated with overall survival after DEB-TACE, stressing the role of post-TACE events, was performed.

**Results:**

The objective response (OR) rate (Modified Response Evaluation Criteria in Solid Tumors (mRECIST) criteria) to the first DEB-TACE (DEB-TACE-1) was 70.3%; the median OS from DEB-TACE-1 was 27 months (95% confidence interval (CI), 24–30). In the multivariate analysis, tumor size, AFP < 100 ng/mL and serum alkaline phosphatase were independent factors for survival following DEB-TACE-1. The most important clinical event associated with poor survival was the development of early ascites after DEB-TACE-1 (median OS, 17 months), which was closely related to the history of ascites, albumin and hemoglobin but not to tumour load or to response to therapy.

**Conclusions:**

Early ascites post-DEB-TACE is associated with the survival of patients despite adequate liver function and the use of a supra-selective technical approach. History of ascites, albumin and hemoglobin are major determinants of the development of early ascites post-DEB-TACE.

## Background

Hepatocellular carcinoma (HCC) is the sixth most common cancer worldwide and is the fourth-leading cause of cancer-related mortality [1, 2]. According to the European and American guidelines [3, 4], transarterial chemoembolization (TACE) is the first-line treatment for asymptomatic patients with Barcelona Clinic Liver Cancer (BCLC) B stage disease (which includes multinodular HCC beyond the Milan criteria, without portal invasion or extrahepatic disease) and compensated liver function. TACE is performed not only in BCLC-B patients but also in early-stage patients if resection, ablation or liver transplantation is not feasible. Thus, TACE candidates represent a heterogeneous group of patients with variable tumour burden and liver function [5].

TACE is an image-guided transcatheter tumour therapy that has an ischaemic and cytotoxic effect on tumour tissue. The use of drug-eluting embolic chemoembolization (DEB-TACE) is safe and effective [6, 7]. However, an improvement in the overall survival (OS) of DEB-TACE compared to that of conventional TACE has not been confirmed [8, 9].

Considering the heterogeneity of TACE candidates, patient selection must be carefully carried out. Underlying chronic liver disease is exacerbated by this procedure, especially in patients with diminished liver reserve [10]. Several algorithms have been recently reported to predict HCC prognosis in an attempt to optimize chemoembolization treatments, but few data have been obtained from DEB-TACE procedures [11–13]. Moreover, there is also scarce information about the influence of post-DEB-TACE events on OS and hepatic decompensation.

A prior meta-analysis of untreated patients in randomized clinical trials for HCC reported that ascites is strongly linked to a worse outcome in intermediate/advanced BCLC stages [14].

Some authors have suggested that a time-dependent covariate analysis that includes all the rounds of DEB-TACE, clinically relevant events and subsequent therapies is needed to properly evaluate the factors that influence the survival of patients.

The aim of our study was to identify predictive factors for survival in HCC patients treated with DEB-TACE, taking into account the basal characteristics, the procedure, the response to treatment and the impact of events after the first DEB-TACE (DEB-TACE-1), in a time-dependent covariate analysis.

## Methods

### Patients

From October 2008 to October 2015, patients with HCC diagnosed according to the European Association for the Study of the Liver (EASL) guidelines who were selected for DEB-TACE were referred to a tertiary academic university hospital and were prospectively registered.

The inclusion criteria were as follows: 1) HCC that was diagnosed in the early stage but was not eligible for resection, ablation or liver transplant; 2) HCC with an intermediate BCLC stage; 3) compensated cirrhosis with normal or mildly altered liver function, without ascites or encephalopathy at the time of DEB-TACE; 4) an asymptomatic status, with an ECOG performance status 0; and 5) approval for DEB-TACE after evaluation by the multidisciplinary tumour board. Portal thrombosis, impaired liver function, current decompensated cirrhosis, performance status > 0, extrahepatic disease and contraindication or impossibility for catheterization or chemoembolization were considered exclusion criteria. Patients included in clinical trials or awaiting liver transplantation for whom DEB-TACE was used as a bridge therapy were excluded.

Clinically significant portal hypertension (CSPH) was defined as the presence of prior cirrhosis decompensation, oesophageal or gastric varices or low platelet counts (lower than 100 ×  10^9^/L) [15] and early ascites as the appearance of ascites after the first round of DEB-TACE.

Clinical, biochemical and radiological examinations were performed at baseline and prior to every DEB-TACE procedure. No general sedation was used, and no antibiotic prophylaxis was indicated, except in patients with prior endoscopic retrograde cholangiopancreatography. If the prothrombin rate was lower than 50% or if the platelet count was less than 50 × 10^9^/L, fresh-frozen plasma was administered and/or platelet infusion was performed. Pain during the procedure was individually managed, and patients were discharged 24 h later, unless complications were observed.

### DEB-TACE procedure

Drug-eluting beads® were loaded with doxorubicin following the manufacturer’s instructions the day before the procedure. Particles that were 300–500 μm (μm) were used until March 2013, when these particles were replaced by 100–300-μm beads to further penetrate the tumour [16]. If embolization was not completely achieved unloaded microspheres were employed to complete the artery obstruction.

Selective angiography of the common hepatic artery was carried out as well as of the right and left hepatic arteries. A supraselective approach for tumour vessels was achieved by using a Progreat 2.7 (Terumo®) microcatheter with 0.21, 0.16 or 0.14 Terumo® microwires, and DC-Beads® were then injected. After angiographic control, the 4F catheter and the introducer were removed, and manual compression was applied.

Starting in February 2015, Cone-Beam-CT software (CBCT, Syngo DynaCT, Siemens®) with contrast injection was employed to help during vascular catheterization, especially if the nodule was not visible at basal angiography or was in intersegmental nodules, when lesions were proximal to the diaphragm and when extrahepatic vascularization was evaluated. Once the procedure was finished, CBCT without intraarterial contrast was used to evaluate embolization.

### Follow-up

All patients received a clinical, analytical and radiological follow-up 6 weeks after each DEB-TACE procedure. Response to treatment was evaluated by contrast-enhanced computed tomography (CT) according to the Modified Response Evaluation Criteria in Solid Tumors (mRECIST) criteria [17], and results were presented to the multidisciplinary tumour board. If partial response, stable disease or treatable progression were observed, a subsequent DEB-TACE was planned [18]. Patients with untreatable progression were evaluated for systemic therapy. Objective response (OR) was defined as the sum of complete response and partial response. The disease control rate (DCR) was defined as the OR as well as the stable disease rate.

### Statistical analysis

Quantitative variables are expressed as the median and interquartile range, and categorical variables are expressed as the count and proportion. Continuous quantitative variables were categorized according to the median value for the analysis. Differences between subgroups were evaluated with a Chi-squared test, Fisher’s exact test and U-Mann Whitney, depending on the type of variable. A conventional *p*-value of less than 0.05 was considered significant.

Patient survival probability was estimated using the Kaplan-Meier method. OS was calculated from DEB-TACE-1 to death or to the end of follow-up for two periods: **from baseline (t**_**0**_**)**, taking into account clinical, demographic and radiological data prior to DEB-TACE-1, **and from 6 weeks after DEB-TACE-1 (t**_**1**_**)**, also considering complications and the radiological response to treatment. Variables with univariate significance (*p* <  0.10) and clinical relevance were included in the Cox proportional hazards model for the multivariable analysis with the forward selection method.

The factors associated with the development of early ascites were analysed, bearing in mind the baseline characteristics, the response to treatment and other complications. The time was censored at ascites development, death or the second DEB-TACE (DEB-TACE-2).

All calculations were performed with SPSS version 23 (SPSS Inc., Chicago, IL).

An additional time-dependent covariate analysis was performed by using **R (****www.r-project.org****)** to identify factors associated with mortality. A backward method based on the Akaike information criteria was employed.

The protocol conformed to the ethical guidelines of the 1975 Declaration of Helsinki and was approved by the Ethics Committee of the Hospital Universitario Central de Asturias (Approval No. 120/19). This prospective database has been retrospectively reviewed and because of the retrospective nature of the study consent retrieval was waived.

## Results

From October 2008 to October 2015, 242 consecutive patients with HCC who were diagnosed according to EASL guidelines were referred for DEB-TACE, but only 216 of these patients met the inclusion criteria. Table 1 summarizes the baseline characteristics. The predominant aetiology of liver disease was alcohol (45%), followed by hepatitis C virus infection (36%). Most of the patients were BCLC-B (58%) and Child-Pugh class A 5 (64%). Sixty-one percent had oesophageal varices, 32% were on a low-salt diet and / or diuretic and 73% presented CSPH.

**Table 1:** Baseline characteristics of the patients (*n* = 216)

Age (yr), median (range), IQR	70 (38–84), 63–76
Gender (Male/Female), n (%)	180 (83)/36 (17)
Alcohol/HCV/other etiologies, n (%)	97 (45)/ 78 (36)/ 41 (19)
Prior radiological ascites (no / yes), n (%)	198 (92) / 18 (8)
Diuretic treatment (no/yes, not available), n (%)	133 (62)/67 (31)/16 (7)
Esophageal varices (yes/no/not available), n (%)	72 (33)/131 (61)/13 (6)
Child-Pugh (A5/A6/B/ not available), n (%)	139 (64)/39 (19)/18 (7.4)/20 (9.6)
BCLC-B (0/A/B), n (%)	10 (5)/77 (37)/129 (58)
Bilirubin (mg/dL), median (range), IQR	1 (0.2–5.4), 0.93–1.33
Albumin, (g/L), median (range), IQR	40 (27–49), 36–43
AFP (ng/mL), median (range), IQR	12.8 (0.3–13-000), 4.9–60.3
Cr (mg/dL), median (range), IQR	0.84 (0.43–3.96), 0.72–0.99
Sodium (mEq/L), median (range), IQR	141 (130–146), 138–142
AST (IU/L), median (range), IQR	50 (15–285), 32–84
ALT (IU/L), median (range), IQR	38 (7–278), 25–76.5
GGT (IU/L), median (range), IQR	120 (16–2011), 67–212
AP (IU/L), median (range), IQR	108 (36–370), 86.5–138.5
PT (%), median (range), IQR	84 (36–118), 75–94
Platelets (× 10^9^/L), median (range), IQR	113 (22–460), 79–159
Hemoglobin (g/dL), median (range), IQR	13.6 (7.6–17.9), 12.4–14.9
Clinically Significant Portal Hypertension (yes/no), n	157 (73) / 59 (27)
Main nodule diameter (mm), median (range), IQR	35 (11–100), 23–48
Previous treatment (ablation/resection), n (%)	44 /8
ALBI 1/2/3/not available, n	81 /96/1/38
Beads size 300–500 μm/ 100–300 μm, n	135/81
Use of Cone Beam CT (no/yes), n	187/29
Dose of doxorubicin (mg), median (range), IQR	90 (7.5–150), 70–140

*Yr* Year, *IQR* Interquartile range, *HCV* Hepatitis C virus, *BCLC* Barcelona Clinic Liver Cancer, *AFP* Alpha-fetoprotein, *Cr* Serum creatinine, *AST* Aspartate aminotransferase, *ALT* Alanine aminotransferase, *GGT* Gamma-glutamyl-transpeptidase, *AP* Alkaline phosphatase, *PT* Prothrombin time

**Fig. 1:**
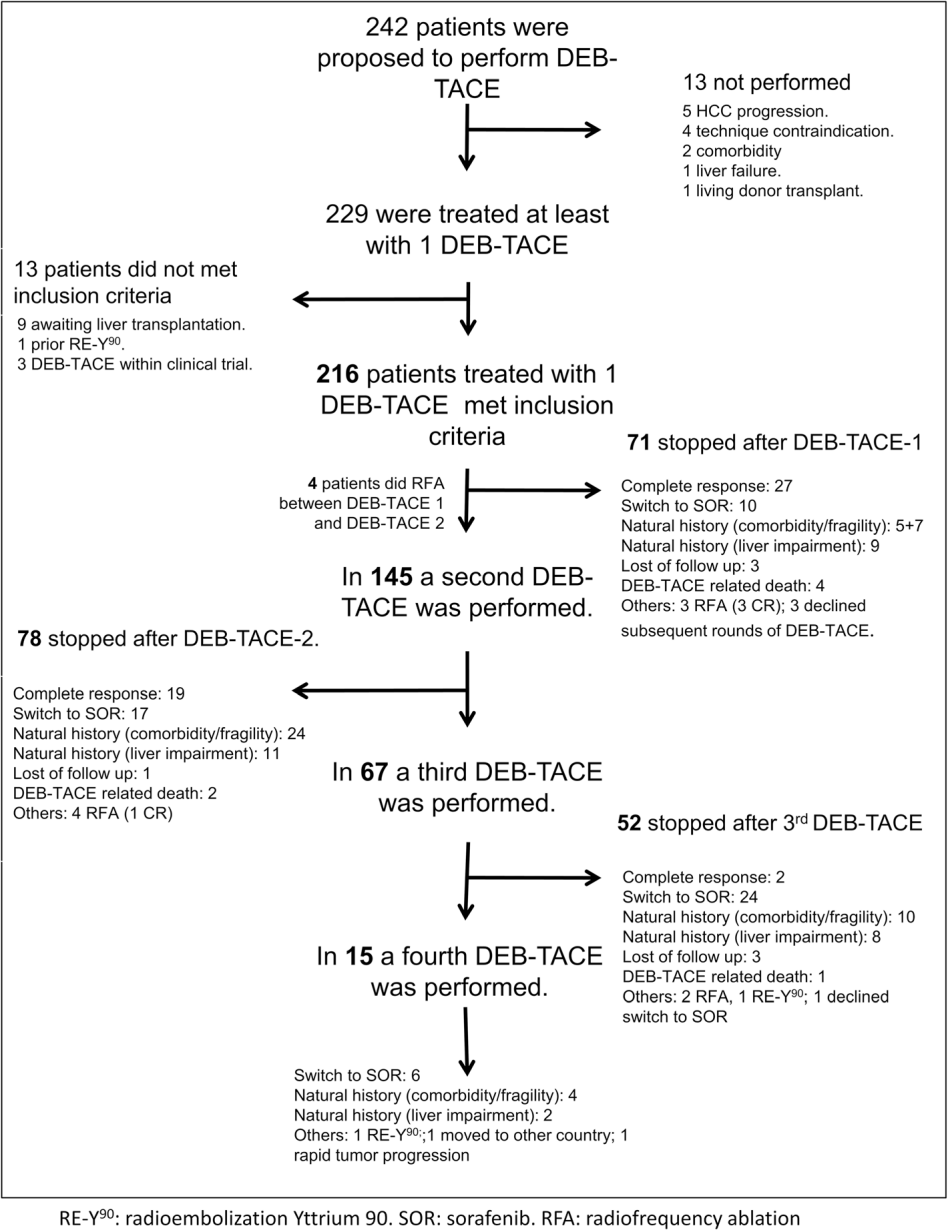
Flowchart of patients enrolled in the study

The median number of DEB-TACE sessions was 2 (IQR, 1–3), and a total of 443 procedures were performed (Fig. 1).

The median follow-up was 26.5 months, and follow-up was censored at death, loss to follow-up or the last visit (April 1, 2019).

### Response to treatment

According to the mRECIST criteria at week 6 after DEB-TACE-1, complete response was achieved in 55 patients (26%), partial response in 97 patients (45%) and stable disease in 24 (11%). In contrast, 35 patients (16%) presented progressive disease after DEB-TACE-1. 35% of those migrated to BCLC-C stage. In 5 patients (2%), mRECIST was not available.

### Follow-up and post-DEB-TACE events

During the follow-up, DEB-TACE was discontinued in 71 patients after the first session. Figure 1 shows the different causes for the discontinuation of DEB-TACE after the first or subsequent rounds. Post-procedure events within the first 90 days after DEB-TACE-1 are described in Supplementary Table 1. A total of 73 of 216 patients experienced post-DEB-TACE-1 events (33.7%): 23 patients (31.5%) experienced radiological events, 41 patients (56.2%) experienced clinical events and 9 patients (12.3%) experienced both clinical and radiological events. Ascites was the most frequent adverse event and was present in 27 patients.

At the end of follow-up, 27 patients were alive; 70 patients had moved to sorafenib and 9 to second-line regorafenib.

### Overall survival

**Fig. 2:**
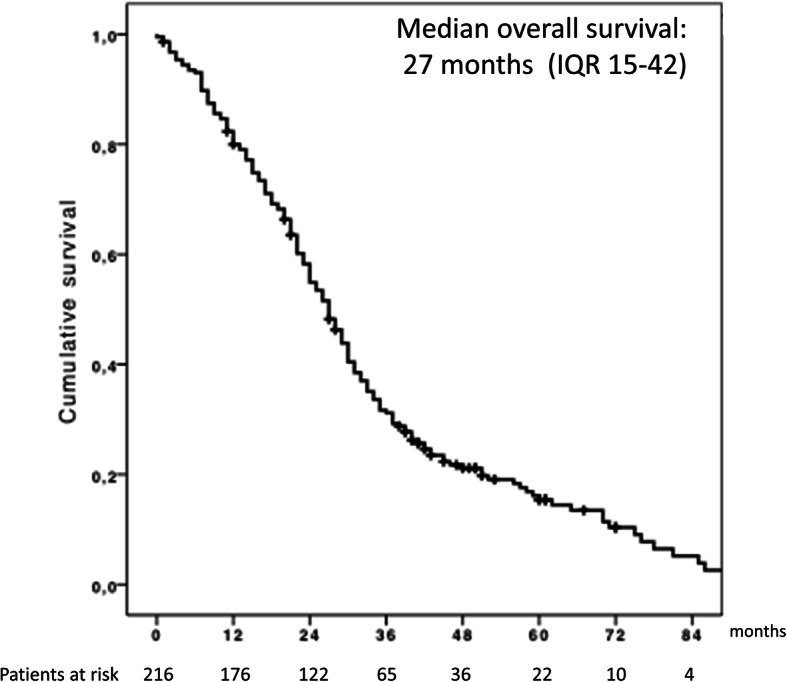
Kaplan-Meier graph with the overall survival of the cohort

The median OS from DEB-TACE-1 was 27 months (95% confidence interval (CI), 24.193–29.807) (Fig. 2). The cumulative survival rates were 82, 58, 32 and 16% at 1, 2, 3 and 5 years, respectively.

. Univariate and multivariate analyses prior to DEB-TACE-1 (**t**_**0**_) and at 6 weeks after DEB-TACE-1 (**t**_**1**_) are shown in Tables 2 and 3. Supplementary Table 2 collects the variable values in **t1**.The independent factors associated with OS at **t**_**0**_ were tumor size < 36.5 mm (cut-off estimated by AUROC) 30 vs 22 months HR 0.72 (95% CI 0.53–0.98, *p* = 0.039), basal AFP < 100 ng/mL (arbitrary value) 29 vs 18 months HR 0.67 (95% CI 0.47–0.96, *p* = 0.029) and basal alkaline phosphatase < 108 IU/L (median value) 32 vs 24 months HR 0.64 (95% CI 0.47–0.88, *p* = 0.005). By contrast, the independent factors associated with OS at **t**_**1**_ were post-TACE albumin < 35 g/L (arbitrary cut-off) 22 vs 30 months HR 1.5 (95% CI 1.1–2.2, *p* = 0.02), post TACE AFP < 100 ng/mL (arbitrary cut-off) 29 vs 12 months HR 0.65 (95% CI 0.45–0.93, *p* = 0.02) and absence of development of ascites (28 vs 17 months, HR 0.43, 95% CI 0.27–0.68, *p* <  0.001). There were four additional models, 2 of them including objective response, detailed in Table 3.

**Table 2:** Predictors of overall survival from DEB-TACE-1 (period t_0_) with pre-DEB-TACE variables (*n* = 216) based on multivariate Cox regression

T_**0**_			Univariate analysis			Multivariate analysis		
**Variable**	**Categories**	***n*** **= 216**	**Overall survival (months)**		***p*-value**	**HR**	**HR 95% CI**	***p*-value**
			median	median 95% CI				
Esophageal varices	No varices	72	30	22.7–37.2	0.025			
	Varices	131	25	21.2–28.8				
> 3 nodules	No	180	27	23.8–30.2	0.029			
	Yes	33	23	18.6–27.4				
**Tumor size**^b^**(mm)**	< 36.5	128	30	26.9–33.1	**0.025**	**0.72**	**0.53–0.98**	**0.039**
	> 36.5	84	22	17.5–26.5				
AFP pre-TACE ^a^ (ng/mL**)**	< 12.8	105	30	25.8–34.2	0.008			
	> 12.8	105	23	19.2–26.8				
**AFP pre-TACE (ng/mL)**	< 100	165	29	26.1–31.8	**0.009**	**0.67**	**0.47–0.96**	**0.029**
	> 100	44	18	13.4–22.6				
AFP pre-TACE (ng/mL**)**	< 200	181	29	26.5–31.5	< 0.001			
	> 200	28	15	11.1–18.9				
AFP pre-TACE (ng/mL**)**	< 400	192	28	25.2–30.8	0.002			
	> 400	17	13	8.2–17.8				
Bil pre-TACE ^a^ (mg/dL)	< 1	130	28	24.2–31.8	0.052			
	> 1	79	25	21.3–28.7				
Bil pre-TACE (mg/dL)	< 2	191	27	23.8–30.1	0.47			
	> 2	17	27	23.1–30.9				
Alb pre-TACE ^a^ (g/L)	< 40	110	24	20.3–27.3	0.112			
	> 40	98	28	24.34–31.6				
Alb pre-TACE (g/L)	< 35	39	23	18.6–27.4	0.021			
	> 35	169	29	25.9–32.04				
**AP preTACE**^a^**(IU/L)**	< 108	105	32	28.01–35.9	< 0.001	0.64	0.47–0.88	0.005
	> 108	105	24	20.8–27.2				
Hb pre-TACE^a^ (g/dL)	< 13.6	108	24	19.02–28.9	0.155			
	> 13.6	105	28	25.6–30.3				
Platelets preTACE, 10^^9^ / L	< 100	90	26	22.3–29.6	0.93			
	> 100	121	27	22.7–31.2				
Ascites preTACE	No	198	27	24.4–29.6	0.068			
	Yes	18	21	16.9–25.01				
CSPH	No	59	30	22.5–37.5	0.045			
	Yes	157	26	22.3–29.7				

*CI* Confidence interval, *HR* Hazard ratio, *TACE* Transarterial chemoembolization, *BCLC* Barcelona Clinic Liver Cancer, *CSPH* Clinically significant portal hypertension., *AFP* Alpha-fetoprotein, *Bil* Bilirubin, *Alb* Albumin, *AP* Alkaline phosphatase, *Hb* Hemoglobin

^a^The median values were used as a cut-off for continuous variables. ^b^ Tumor size estimated by AUROC

Other variables evaluated: sex (*p* = 0.156), etiology (0.197); diabetes (*p* = 0.929); AST (*p* = 0.340); ALT (*p* = 0.791); GGT (*p* = 0.289); Creatinine (*p* = 0.847); Na (*p* = 0.944); CBCT use (*p* = 0.495); DEB size (*p* = 0.283)

**Table 3:** Predictors of overall survival in the t_1_ period (from DEB-TACE-1 assessment) including pre and post-procedure variables (*n* = 216) based on multivariate Cox regression

T_**1**_			Univariate analysis			Multivariate analysis		
**Variable**	**Categories**	***n*** **= 216**	**overall survival, months**		***p*-value**	**HR**	**HR, 95% CI**	***p*-value**
			median	median, 95% CI				
Number nodules	< 3	165	27	23.2–30.8	0.07			
	> 3	51	24	18.4–26.9				
**Main nodule size**^b^**, mm**	< 36.5	110	30	26.5–33.5	**0.005**	0.59	0.45–0.84	0.003
	> 36.5	103	22	18.9–25.03		0.65	0.46–0.92	0.013
EV	No	72	30	22.8–37.2	**0.03**			
	Yes	144	26	22.8–29.2				
CSPH	No	61	30	24.9–35.1	**0.05**			
	Yes	153	25	21.4–28.6				
Gender	Female	37	31	21.5–40.5	0.14			
	Male	179	27	23.8–30.1				
**Albumin preTACE**^a^**(g/L)**	< 40	110	24	20.3–27.7	0.2	0.61	0.4–0.93	0.023
	> 40	98	28	24.1–31.8				
**AP preTACE**^a^**(IU/L)**	< 108	105	32	28.01–35.9	**< 0.001**	0.65	0.44–0.94	0.02
	> 108	104	24	20.7–27.2		0.56	0..39–0.79	0.001
						0.54	0.38–0.76	0.001
						0.59	0.43–0.8	0.001
**AFP preTACE**^a^**(ng/mL)**	< 12.8	105	30	25.7–34.2	**0.009**	0.66	0.46–0.94	0.002
	< 12.8	104	23	19.3–26.7		0.7	0.49–0.98	0.04
**Ascites preTACE**	No	198	28	25.3–30.6	0.06	0.39	0.19–0.76	0.006
	Yes	18	20	16.1–23.9				
**Doxorubicin dose**^a^ (mg)	< 90	95	29	25.4–32.5	**0.03**			
	> 90	94	24	20.5–27.6				
CBCT	No	187	27	23.7–30.3	0.25			
	Yes	29	24	19.4–28.6				
Particle size, μm	300–500	135	26	23.2–28.8	0.53			
	100–300	81	29	23.6–34.4				
**Post-TACE-1 events**	No	143	29	26.3–31.6	**0.005**			
	Yes	73	22	16.3–27.7				
Hb post-TACE-1 ^a^ (g/dL)	< 13.4	109	26	20.2–31.8	0.83			
	> 13.4	98	28	24.3–31.7				
Platelets post-TACE-1^a^ ×10^^9^/L	< 108.5	103	26	21.4–30.6	0.66			
	> 108.5	103	27	22.4–31.5				
Platelets post-TACE-1 × 10^^9^/L	< 100	87	26	21.3–30.7	0.75			
	> 100	119	27	22.7–31.2				
**Albumin post-TACE-1**^a^**(g/dL)**	< 38	106	21	18.7–23.3	**< 0.001**	2.5	1.6–3.9	< 0.001
	> 38	102	35	28.8–41.2				
**Albumin pos-TACE 1 g/dL**	< 35	54	22	18.5–25.5	**0.001**	1.5	1.1–2.2	0.02
	> 35	154	30	26.9–33.1				
∆ Albumin g/dL	< 0	138	25	21.7–28.3	0.07			
	0	22	37	27.5–46.5				
	> 0	40	29	24.8–33.1				
AFP post-TACE 1^a^ ng/mL	< 8.4	105	30	25.4–34.5	**0.025**			
	> 8.4	104	24	20.5–27.5				
**AFP post-TACE 1 ng/mL**	< 100	183	29	26.4–31.6	**< 0.001**	0.65	0.45–0.93	0.02
	> 100	26	12	8.3–15.7		0.67	0.47–0.97	0.03
AFP post-TACE 1 ng/mL	< 200	192	29	26.5–31.5	**0.005**			
	> 200	171	14	9.9–18.03				
AFP post-TACE 1 ng/mL	< 400	159	29	26.5–31.5	**< 0.001**			
	> 400	13	12	7.3–16.7				
∆ AFP ng/mL	< 0	139	28	24.7–31.3	0.23			
	0	6	14	5.6–22.4				
	> 0	57	27	22.1–31.8				
Bilirubin post-TACE 1^a^ mg/dL	< 1	140	30	25.4–34.5	**0.01**			
	> 1	71	24	20.6–27.4				
Bilirubin pos. TACE 1 mg/dL	< 2	194	28	25.3–30.7	**0.03**			
	> 2	17	15	5.6–24.4				
∆ Bilirubin mg/dL	< 0	74	27	21.4–32.6	0.003			
	0	62	34	26.1–41.9				
	> 0	67	23	20.1–25.9				
AST post-TACE 1^a^ IU/L	< 47	104	27	22.5–31.5	0.67			
	> 47	100	29	24.7–33.2				
ALT post-TACE 1^a^ IU/L	< 35	104	26	22.7–29.2	0.5			
	> 35	103	29	25.8–32.2				
GGT post-TACE 1^a^ IU/L	< 133	105	29	21.7–36.3	0.23			
	> 133	100	27	24.1–29.8				
**AP post-TACE 1**^a^ IU/L	< 128	102	33	28.3–37.6	**< 0.001**			
	> 128	102	20	11.6–28.3				
∆ AP post-TACE 1 IU/L	< 0	57	30	23.7–36.3	0.66			
	0	2						
	> 0	139	27	23.8–30.2				
Creatinine post-TACE 1^a^ mg/dL	< 0.79	104	28	24.2–31.8	0.68			
	> 0.79	101	27	21.4–32.5				
Sodium post-TACE 1^a^ mEq/L	< 140	105	26	22.5–29.5	0.28			
	> 140	102	29	24.6–33.4				
PT post-TACE 1^a^ %	< 82	106	27	22.9–31.1	0.21			
	> 82	102	28	22.8–33.1				
**Ascites post-TACE 1**	No	189	28	25.5–30.5	**< 0.001**	0.41	0.25–0.7	0.001
	Yes	27	17	8.6–25.4		0.38	0.24–0.63	< 0.001
						0.43	0.27–0.68	< 0.001
						0.37	0.23–0.58	< 0.001
Progressive disease to TACE-1 (mRECIST)	No	176	28	25.5–30.5	**0.03**			
	Yes	35	23	20.8–25.2				
RECIST	CR	55	28	20.8–35.1	**< 0.001**			
	PR	97	29	26.1–31.8				
	SD	24	26	18.04–33.9				
	PD	35	23	20.1–25.2				
	Not available	5	2	0–4.2				
**Objective response**	No	64	23	20.2–25.8	**0.01**	0.67	0.46–0.98	0.04
	Yes	152	29	26.3–31.7		0.68	0.47–0.99	0.048

The model 4 uses only three variables, all of them post-TACE, with significant thresholds in two variables (albumin 35 g/L, AFP 100 ng/mL) and appearance of ascites

*CI* Confidence interval, *HR* Hazard ratio, *TACE* Transarterial chemoembolization, *BCLC* Barcelona Clinic Liver Cancer, *CSPH* Clinically significant portal hypertension., *AFP* Alpha-fetoprotein, *Bil* Bilirubin, *Alb* Albumin, *AP* Alkaline phosphatase, *Hb* Hemoglobin, *CBCT* Cone-beam computed tomography, *EV* Esophageal varices. ^a^ The median values were used as a cut-off for continuous variables. ^b^ Tumor size estimated by AUROC. ∆ Bil, ∆ AP, ∆ AFP, and ∆ alb are calculated by the subtraction of pre-TACE from post-TACE variables

**In the T1 period** multivariate analysis includes basal (preTACE-1) variables statistically significant and those considered clinically relevant, together with significant variables of T1 period. Five models have been developed

**Model 1**: basal albumin < 40 g/L (median value), basal AP < 108 IU/L (median value), ascites preTACE, albumin posTACE < 38 g/L (median value) and ascites posTACE

**Model 2**: tumor size < 36.5 mm (cut-off estimated by AUROC), basal AFP < 12.8 ng/mL (median value), basal AP < 108 IU/L (median value) and objective response

**Model 3**: tumor size < 36.5 mm (estimated by AUROC), basal AFP < 12.8 ng/mL (median value), basal AP < 108 IU/L (median value), objective response and ascites posTACE

**Model 4:** albumine postTACE < 35 g/L (arbitrary), AFP < 100 ng/mL postTACE (arbitrary) and ascites posTACE

**Model 5**: basal AP < 108 IU/L (median), AFP < 100 ng/mL postTACE (arbitrary) and ascites posTACE

### Post-DEB-TACE events

**Fig. 3:**
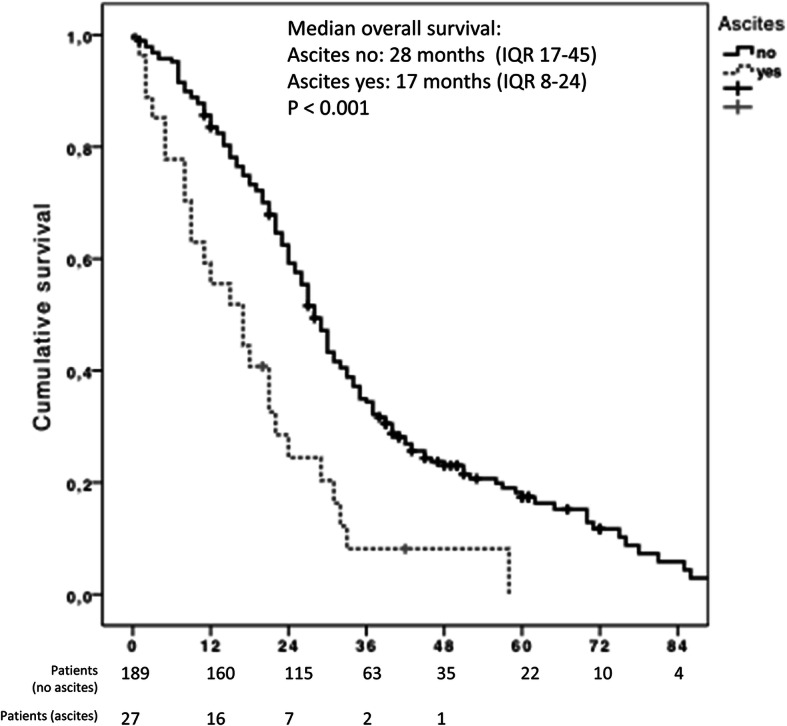
Kaplan-Meier graph with the overall survival according to the development of ascites

The most important clinical event associated with shorter survival was the presence of ascites after DEB-TACE-1 (Fig. 3). Patients with ascites (*n* = 27) had a median OS of 17 months (95% CI, 8.566–25.434; *p* <  0.05); in contrast, patients without ascites had a median OS of 28 months (95% CI, 25.519–30.481). Cumulative survival rates at 1, 2, 3 and 5 years are shown in Fig. 3.

Baseline characteristics associated with the development of ascites are shown in Supplementary Table 3. History of ascites decompensation, haemoglobin and albumin were independently related to the development of early ascites (Table 4). The development of ascites after DEB-TACE was independent of the radiological response (the OR rate was similar in patients who developed ascites, indicating that this was not related to tumour progression, *p* = 0.13). Lastly, 9 patients recovered from hepatic decompensation and received DEB-TACE-2. The median time between decompensation and TACE retreatment was 52 days (range 181, IQR 46.5–132).

**Table 4:** Predictors of development of early ascites based on multivariate Cox regression, censoring the time of follow-up at death, early ascites, or second DEB-TACE

	*p*-value	Exp(B)	Exp(B) 95% CI	
			Lower	Higher
CSPH (yes/no)	0.35	0.34	0.05	2.99
Esophageal varices (yes/no)	0.43	0.61	0.1	2.10
BCLC 0/ A (yes/no)	0.20	1.80	0.730	4.46
Bilirubin	0.15	1.53	0.85	2.74
Hemoglobin	0.01	0.66	0.49	0.89
Child A5 (yes / no)	0.001	0.22	0.09	0.53
Albumin	0.03	0.31	0.11	0.88
Ascites prior to DEB-TACE	< 0.001	0.12	0.04	0.32

**Model 1**: Child A 5 plus Hemoglobin; **Model 2:** Hemoglobin plus prior ascites plus albumin

*CSPH* Clinically significant portal hypertension

In the time-dependent covariate analysis, the variables that were independently associated with survival were basal alpha-fetoprotein (HR, 1.66; 95% CI, 1.31–2.10; *p* <  0.001), time-dependent bilirubin (HR, 4.47; 95% CI, 1.80–11.09; *p* <  0.001) and time-dependent alkaline phosphatase (HR, 1.68; 95% CI, 1.41–2.01; *p* <  0.001) (Supplementary Tables 4 and 5 and Supplementary figure 1).

## Discussion

Several publications have described the safety of DEB-TACE [6–9], but an in-depth analysis of the impact of adverse events on patient progression has not been examined. Our study determined that the development of early ascites was negatively related to the overall survival of compensated patients treated with DEB-TACE. This finding was related to low albumin, low haemoglobin and prior episodes of clinical ascites. The presence of significant portal hypertension and/or worse liver function might suggest that patients are predisposed to complications in chemoembolization procedures and to the consequent impairment in OS. Currently, the presence of CSPH precludes patients from undergoing surgical resection for HCC [19], but less attention has been paid to other loco-regional therapies. However, two studies have recently reported that CSPH is a major negative prognostic factor in patients treated with DEB-TACE [10, 20].

In our clinical practice, patients with previous decompensation that remain compensated for more than 6 months are not excluded for treatment with DEB-TACE. That is the case in 71 patients with alcohol related cirrhosis with a first episode of hepatic decompensation that are asymptomatic and compensated after alcohol withdrawal. It should be noted that the OS of this cohort was lower than that at other sites [21–29] (Supplementary Table 6), despite similar patient selection, supra-selective procedures and response to therapy. We speculate that although alcohol aetiology is not an independent predictor of survival, alcohol consumption can impair liver function due to acute-on-chronic liver failure [30] or alcoholic hepatitis [31, 32]. The poor prognosis of alcohol-related HCC has been specifically observed in some French cohort studies [33, 34] and in patients treated with Y^90^-radioembolization in the SORAMIC study [35]. Indeed, these patients had more comorbidities than those affected by hepatitis C, and in some cases, refused to undergo additional sessions of DEB-TACE. In our series, 6 patients presented a second primary tumour after DEB-TACE (1 pyriform sinus, 1 bladder, 1 colorectal and 3 lung cancers), and 13% of patients died from other causes that were not related to tumour progression or cirrhosis decompensation. Finally, some post-DEB-TACE events were handled out of the tertiary hospital, and the suboptimal care of cirrhosis complications could have influenced the survival of our patients.

Alkaline phosphatase has resulted as independent factors for survival following DEB-TACE-1, together with tumor size and AFP. This enzyme is a variation marker for embryonic stem cell and could indicate the proliferation of tumor cells, playing an important role in cell cycle regulation, cell proliferation and tumor formation. High levels of AP have been related to poor prognosis of HCC in different populations [36, 37].

This study had several weaknesses. This was an observational cohort study that was performed over many years, during which changes in the state-of-the-art technology occurred. However, the multidisciplinary core team and the main interventional radiologists did not change over the course of the study. Furthermore, neither the change in the DEB particles size (March 2013) nor the introduction of CBCT (February 2015) have influenced the objective response rate or the global overall survival (data not shown).

The second weakness was that no clinical events were identified in the time-dependent covariate analysis.

In contrast, the main advantage of this study was the prospective collection of a large number of patients who were treated with a homogeneous protocol and the collection of adverse effects after DEB-TACE, including asymptomatic radiological abnormalities.

## Conclusions

In conclusion, adverse events reduce OS following DEB-TACE, especially when ascites is present. Although compensated chronic liver disease is a requirement for loco-regional therapy, the appearance of early ascites seems to be related to the history of prior ascites, lower haemoglobin levels and lower albumin. These factors could be relevant for properly selecting the best candidates for DEB-TACE when different therapeutic options are available.

## Supplementary information

**Additional file 1 Supplementary Figure 1**. probability of death in an average individual.

**Additional file 2 Supplementary Table 1**. Post-DEB-TACE events.

**Additional file 3 Supplementary Table 2**. Variable values in the pos-TACE time (t1).

**Additional file 4 Supplementary Table 3**. Baseline characteristics associated with the development of early ascites.

**Additional file 5 Supplementary Table 4.** Time dependent-covariate analysis. Univariate model.

**Additional file 6 Supplementary Table 5**. Time-dependent multivariate analysis.

**Additional file 7 Supplementary Table 6**. Cohort studies on TACE/DEB-TACE focused on alcohol etiology and survival.

## Data Availability

The datasets used and/or analysed during the current study are available from the corresponding author on reasonable request.

## Abbreviations

AFP: Alpha-fetoprotein
AP: Alkaline phosphatase
BCLC: Barcelona Clinic Liver Cancer
CBCT: Cone-beam computed tomography
CI: Confidence interval
CT: Computed tomography
CR: Complete response
CSPH: Clinically significant portal hypertension
CDR: Disease control rate
DEB-TACE: Drug-eluting embolic chemoembolization
EASL: European Association for the Study of the Liver
HCC: Hepatocellular carcinoma
HR: Hazard ratio
IQR: Interquartile range
mRECIST: Modified Response Evaluation Criteria in Solid Tumors
OR: Objective response
OS: Overall survival
