# Supplementary material for: The development of early ascites is associated with shorter overall survival in patients with hepatocellular carcinoma treated with drug-eluting embolic chemoembolization
Source: BMC Gastroenterol. 2020 Jun 1;20:166. doi: 10.1186/s12876-020-01307-x (PMC7268728; doi:10.1186/s12876-020-01307-x)
Supplement: Supplementary file 2 — Additional file 2 Supplementary Table 1. Post-DEB-TACE events. [file 12876_2020_1307_MOESM2_ESM.docx]

**Supplementary table 1:** Post-DEB-TACE events.

| **Post-TACE events (n, patients)** | **Overall survival from DEB-TACE 1 (months), median (95% CI)** |
| --- | --- |
| No post-DEB TACE events (143) | 29 (26.5-31.5) |
| **Radiological (23)**  . Portal thrombosis (12)  . Arterial dissection (3)  . Biliary dilation (7)  . Intratumoral bleeding (1) | 27 (20.7-33.3) |
| **Clinical (41)**  Non-cirrhotic complications (19)  . Cholecystitis (1)  . Abscess (1)  . Inguinal hematoma (6)  . Acute myocardial infarction (1)  . Readmission/prolonged admission (4)  . Post-TACE syndrome (3)  . Rash (1)  . Other (2) | 27 (0.3-53.7) |
| Cirrhotic complications  . Hepatic encephalopathy (4)  . Ascites (10)  . Clinical event+ ascites (8) ^Ɨ^ | 34 (0 – 83)  11 (0-23.4)  17 (12.8-21.2) |
| **Radiological + clinical event (9)**  . Radiological+ ascites (8)  . Clinical+ radiological+ ascites (1) | 5 (0-22.5)  9 |
| Global | 27 (24.2 – 29.8) |

^Ɨ^ liver failure (2), readmission (2), abscess (2), cholecystitis (1), radiodermatitis (1).
