# Supplementary material for: The development of early ascites is associated with shorter overall survival in patients with hepatocellular carcinoma treated with drug-eluting embolic chemoembolization
Source: BMC Gastroenterol. 2020 Jun 1;20:166. doi: 10.1186/s12876-020-01307-x (PMC7268728; doi:10.1186/s12876-020-01307-x)
Supplement: Supplementary file 3 — Additional file 3 Supplementary Table 2. Variable values in the pos-TACE time (t1). [file 12876_2020_1307_MOESM3_ESM.docx]

**Supplementary table 2.** Variable values in the pos-TACE time (t1).

|  | **t1** |
| --- | --- |
| Bilirubin (mg/dL), median (range, IQR) | 1 (4.2, 0.96-1.3) |
| Albumin, (g/L), median (range, IQR) | 38 (27, 35-41) |
| AFP (ng/mL), median (range, IQR) | 8.4 (14168.4, 4-28.05) |
| Cr (mg/dL), median (range, IQR) | 0.79 (4.2, 0.7-0.96) |
| Sodium (mEq/L), median (range, IQR) | 140 (23, 138-142) |
| AST (IU/L), median (range, IQR) | 47 (235.8, 33.3-74.7) |
| ALT (IU/L), median (range, IQR) | 35 (239,24-61) |
| GGT (IU/L), median (range, IQR) | 133 (1244, 75-218) |
| AP (IU/L), median (range, IQR) | 128 (422, 93-166) |
| PT (%), median (range, IQR) | 82 (93,74-91) |
| Platelets (x 10^9^/L), median (range, IQR) | 108.5 (355, 78-162.5) |
| Hemoglobin (g/dL), median (range, IQR) | 13.4 (9.6, 12.1-14.3) |
| Post-TACE-events (no/yes), n (%) | 143 (66)/33 (34) |
| Ascites post-TACE (no/yes), n (%) | 189 (88) /27 (12) |
| Child (A5/A6/B/C) | 125 (58)/43(20)/26 (12)/2 (10) |
| MELD: median (range, IQR) | 8 (16, 7-10) |
| ALBI 1/2/3/not available, n (%) | 66 (31)/10 (50)/6 (3)/36 (16) |
| Beads size 300-500 μm/ 100-300 μm, n | 135/81 |
| Use of Cone Beam CT (no/yes), n | 187/29 |
| Dose of doxorubicin (mg), median (range, IQR) | 90 (142.5, 70-140) |

IQR: interquartile range; AFP: alpha-fetoprotein; Cr: serum creatinine; AST: aspartate aminotransferase; ALT: alanine aminotransferase; GGT: gamma-glutamyl-transpeptidase; AP: alkaline phosphatase; PT: prothrombin time.
