# Supplementary material for: The development of early ascites is associated with shorter overall survival in patients with hepatocellular carcinoma treated with drug-eluting embolic chemoembolization
Source: BMC Gastroenterol. 2020 Jun 1;20:166. doi: 10.1186/s12876-020-01307-x (PMC7268728; doi:10.1186/s12876-020-01307-x)
Supplement: Supplementary file 4 — Additional file 4 Supplementary Table 3. Baseline characteristics associated with the development of early ascites. [file 12876_2020_1307_MOESM4_ESM.docx]

**Supplementary table 3:** Baseline characteristics associated with the development of early ascites.

| **Variables**, median (IQR) | **Ascites post-TACE (n=27)** | **No ascites post-TACE (n=189)** | **p-value** |
| --- | --- | --- | --- |
| Prior ascites (no/yes), n | 18/9 | 180/9 | **< 0.001** |
| Esophageal varices (yes/no/unknown), n | 23/4/0 | 108/68/13 | **0.02** |
| CSPH (no/yes), n | 1/ 26 | 58/131 | **0.001** |
| Albumin (g/L) | 38 (33 - 40) | 41 (37 - 43) | **0.01** |
| Bilirubin (mg/dL) | 1.18 (0.97 - 1.75) | 1 (0.9 - 1.25) | **0.06** |
| Child-Pugh (A5/ A6, B), n | 8/9/7 | 131/30/11 | **< 0.001** |
| Hemoglobin (g/dL) | 12.5(11.58 - 13.27) | 13.8 (12.6 - 14.9) | **0.001** |
| BCLC 0/A vs B (yes/no), n | 8 / 19 | 68 / 95 | **0.06** |

IQR: interquartile range; CSPH: clinically significant portal hypertension.

Variables evaluated: age, sex, etiology, diabetes, BCLC-stage, alpha-fetoprotein; aspartate aminotransferase; alanine aminotransferase; gamma-glutamyl-transpeptidase; alkaline phosphatase; prothrombin time; doxorubicin.
