# Supplementary material for: The development of early ascites is associated with shorter overall survival in patients with hepatocellular carcinoma treated with drug-eluting embolic chemoembolization
Source: BMC Gastroenterol. 2020 Jun 1;20:166. doi: 10.1186/s12876-020-01307-x (PMC7268728; doi:10.1186/s12876-020-01307-x)
Supplement: Supplementary file 5 — Additional file 5 Supplementary Table 4. Time dependent-covariate analysis. Univariate model. [file 12876_2020_1307_MOESM5_ESM.docx]

**Supplementary table 3:** Time dependent-covariate analysis. Univariate model

List of time-dependent covariates analyzed:

Start date, end date, varices, prior ascites, BCLC preTACE, AFP_b, Brb_b, Alb_b, AP_b, ALBI_b, ALBI grade_b (b: at baseline); date of DEB-TACE round, Objective response to DEB-TACE, Sorafenib (yes/no), AFP_t, Brb_t, Alb_t, AP_t, ALBI_t, ALBI grade_t (t: time dependent), cirrhosis event (either gastrointestinal bleeding, hepatic encephalopathy or espontaneous bacterial peritonitis), ascites, other complications, death.

Alb: albumin; Brb: bilirubin; AP: alkaline phosphatase.

| Univariate model | HR | 95%CI | p-value |
| --- | --- | --- | --- |
| Ascites  Ascites (No)  Ascites (Yes) | 1 (Ref.)  2.58 | (1.82 – 3.67) | < 0.001 |
| Varices  Varices (No)  Varices (Yes)  Varices (Unknown) | 1 (Ref.)  1.72  1.74 | (1.16 – 2.57)  (0.83 – 3.63) | 0.019  0.007  0.141 |
| Prior ascites  Prior ascites (No)  Prior ascites (Yes) | 1 (Ref.)  1.58 | (0.86 – 2.93]) | 0.143 |
| BCLC_preTACE  (BCLC-0)  (BCLC-A)  (BCLC-B) | 1 (Ref.)  1.81  3.35 | (0.64 – 5.09)  (1.22 – 9.23) | < 0.001  0.261  0.019 |
| AFP_b (per 1000) | 1.16 | (1.03 – 1.30) | 0.014 |
| AFP_b_Categorized  AFP_b_ (Third 1)  AFP_b_ (Third 2)  AFP_b_ (Third 3) | 1 (Ref.)  1.20  1.43 | (0.78 – 1.85)  (0.93 – 2.20) | 0.273  0.408  0.108 |
| Brb_b | 0.96 | (0.69 – 1.33) | 0.792 |
| Alb_b | 1.01 | (0.99 – 1.04) | 0.201 |
| AP_b | 1.00 | (1.00 – 1.00) | 0.222 |
| DEB-TACE (round)  DEB-TACE | 1.03 | (0.85 – 1.26) | 0.716 |
| Objective response to DEB-TACE  OR (No)  OR (Yes)  OR (not assessed) | 1 (Ref.)  0.35  9.78 | (0.21 – 1.58)  (4.50 – 21.25) | < 0.001  < 0.001  < 0.001 |
| Sorafenib  Sorafenib (No)  Sorafenib (Yes) | 1 (Ref.)  0.52 | (0.20 – 0.98) | 0.043 |
| Albi_b_grade  Albi_b_Grade 1  Albi_b_Grade 2  Albi_b_Grade 3 | 1 (Ref.)  1.39  0.70 | (0.98 – 1.97)  (0.10 – 5.11) | 0.159  0.068  0.728 |
| AFP_t (per 1000) | 1.01 | [1.01 – 1.01] | < 0.001 |
| AFP_t_Categorized  AFP_t_ (Third 1)  AFP_t_ (Third 2)  AFP_t_ (Third 3) | 1 (Ref.)  1.17  3.42 | [0.46 – 2.96]  [1.64 – 7.16] | < 0.001  0.736  0.001 |
| Brb_t (per 100) | 3.73 | [1.36 – 10.21] | 0.011 |
| Brb_t_Categorized  Brb_t (Third 1)  Brb_t (Third 2)  Brb_t (Third 3) | 1 (Ref.)  1.85  5.41 | [0.82 – 4.20]  [3.27 – 8.98] | < 0.001  0.141  < 0.001 |
| Albumin_t | 0.45 | [0.33 – 0.62] | < 0.001 |
| AP_t (per 100) | 1.44 | [1.30 – 1.60] | < 0.001 |
| AP_t_Categorized  AP_t_ (Third 1)  AP_t_ (Third 2)  AP_t_ (Third 3) | 1 (Ref.)  1.60  3.86 | [0.83 – 3.12]  [2.16 – 6.93] | <0.001  0.161  <0.001 |
| ALBI_t_categorized  Albi_t_ (Grade 1)  Albi_t_(Grade 2)  Albi_t_ (Grade 3) | 1 (Ref.)  4.60  31.58 | [1.63 – 12.96]  [11.12 – 89.72] | < 0.001  < 0.001  < 0.001 |
| Cirrhosis event  Cirrhosis event (No)  Cirrhosis event (Yes) | 1 (Ref.)  3.22 | [2.07 – 5.02] | < 0.001  < 0.001 |
| Other complications  Other complications (No)  Other complications (Yes) | 1 (Ref.)  1.36 | [0.93 – 1.99] | 0.118  0.111 |

Comment: In this analysis, some of the variables have been categorized by thirds due to the enormous asymmetry and variability observed to try to appreciate some additional significant effect. Thus, in the AFP t it is observed that the risk is 3.42 higher in the third third than in the first, considered as a reference; for AP_t there is a similar result: risk 3.86 times higher in the third third than in the first.
