# Supplementary material for: The development of early ascites is associated with shorter overall survival in patients with hepatocellular carcinoma treated with drug-eluting embolic chemoembolization
Source: BMC Gastroenterol. 2020 Jun 1;20:166. doi: 10.1186/s12876-020-01307-x (PMC7268728; doi:10.1186/s12876-020-01307-x)
Supplement: Supplementary file 6 — Additional file 6 Supplementary Table 5. Time-dependent multivariate analysis. [file 12876_2020_1307_MOESM6_ESM.docx]

**Supplementary table 5:** Time-dependent multivariate analysis.

| **Multivariate model** | **HR** | **95% CI** | **p-value** |
| --- | --- | --- | --- |
| AFP_b (per 1000)   Brb_t_ Categorized     Brb_t_ (Third 1)     Brb_t_ (Third 2)     Brb_t_ (Third 3)   AP_t (per 100) | 1.66    1 (Ref.)  1.49  4.47  1.68 | (1.31 - 2.10)  (0.37 – 5.96)  (1.80 - 11.09)    (1.41 - 2.01) | < 0.001    0.577  < 0.001    < 0.001 |

Comment: In this model it is observed that, significantly, the risk is 66% higher for every 1000 units of baseline AFP and 68% higher for every 100 units of AP_t. Also, it is significant that this risk of mortality is almost 4.5 times higher in the third third of the Brb_t, than in the first third or reference (the confidence interval is relatively broad).
