# Supplementary material for: The development of early ascites is associated with shorter overall survival in patients with hepatocellular carcinoma treated with drug-eluting embolic chemoembolization
Source: BMC Gastroenterol. 2020 Jun 1;20:166. doi: 10.1186/s12876-020-01307-x (PMC7268728; doi:10.1186/s12876-020-01307-x)
Supplement: Supplementary file 7 — Additional file 7 Supplementary Table 6. Cohort studies on TACE/DEB-TACE focused on alcohol etiology and survival. [file 12876_2020_1307_MOESM7_ESM.docx]

**Supplementary table 6:** Cohort studies on TACE/DEB-TACE focused on alcohol etiology and survival.

| **Author, year, ^reference^** | **n** | **Type of TACE** | **Alcohol aetiology (n)** | **Median Overall Survival (months)** |
| --- | --- | --- | --- | --- |
| Malagari; 2012,^21^ | 173 | DEB-TACE | NE | 43.8 |
| Burrell, 2012, ^22^ | 104 | DEB | 22 | 48.6 |
| Kadalayil, 2013,^12^ | 114 | TAE / TACE | Training cohort: 15 | 15 |
|  |  |  | Validation cohort: 42 | 13.7 |
| Terzi, 2014, ^23^ | 51 | cTACE | 6 | 26 |
| Adhoute, 2015, ^13^ | 139 | cTACE | 35 | 28 |
| Chen, 2016, ^24^ | 259 | cTACE | Training cohort: NE | 16.1 |
|  |  |  | Validation cohort: NE | 15.8 |
| Facciorusso, 2016,^25^ | 145  104 | DEB TACE  cTACE | NE  NE | 32 (24-39)  39 (32-47) |
| Pipa-Muniz, 2017,^26^ | 102 | DEB | 40 | 21 |
| Zhang, 2018, ^27^ (Ɨ) | 147 | cTACE | NE | 9 |
| Biolato, 2018, ^28^ | 289 | cTACE/DEB | 9 | 23 |
| Sánchez-Delgado, 2019, ^29^ | 147 | DEB | 22 | 22.75 |

(Ɨ) BCLC-C included. NE: not especified.
